# Melaminium iodide monohydrate

**DOI:** 10.1107/S160053681001785X

**Published:** 2010-05-22

**Authors:** Min Min Zhao, Ping Ping Shi

**Affiliations:** a Ordered Matter Science Research Center, College of Chemistry and Chemical, Engineering, Southeast University, Nanjing 211189, People’s Republic of China

## Abstract

In the title melaminium salt, 2,4,6-triamino-1,3,5-triazin-1-ium iodide monohydrate, C_3_H_7_N_6_^+^·I^−^·H_2_O, the components are linked *via* N—H⋯O, N—H⋯N, O—H⋯I and N—H⋯I hydrogen bonds. All of the H atoms of the melaminium cation are involved in hydrogen bonds. The melaminium cations are inter­connected by four N—H⋯N hydrogen bonds, forming ribbons along [111]. The water mol­ecules connected by N—H⋯O hydrogen bonds also form part of these ribbons. The ribbons are inter­connected by other hydrogen bonds (O—H⋯I and N—H⋯I), as well as by π–π inter­actions [centroid–centroid distance = 3.6597 (17) Å].

## Related literature

For similar singly protonated melaminium salts, see: Janczak *et al.* (2001 ▶); Athikomrattanakul *et al.* (2007 ▶). For ferroelectric materials, see: Fu *et al.* (2009 ▶); Hang *et al.* (2009 ▶). For impedance studies, see: Uthrakumar *et al.* (2008 ▶).
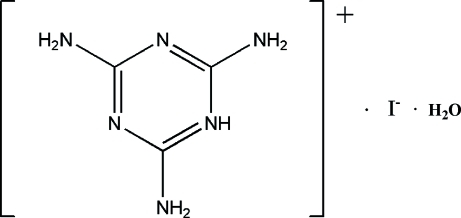


## Experimental

### 

#### Crystal data

C_3_H_7_N_6_^+^·I^−^·H_2_O
*M* *_r_* = 272.06
Triclinic, 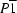

*a* = 6.0655 (12) Å
*b* = 7.0370 (14) Å
*c* = 11.413 (2) Å
α = 104.02 (3)°
β = 93.95 (3)°
γ = 109.08 (3)°
*V* = 440.80 (19) Å^3^
*Z* = 2
Mo *K*α radiation
μ = 3.59 mm^−1^
*T* = 293 K
0.40 × 0.30 × 0.20 mm

#### Data collection

Rigaku SCXmini diffractometer
Absorption correction: multi-scan (*CrystalClear*; Rigaku, 2005 ▶) *T*_min_ = 0.285, *T*_max_ = 0.487
4551 measured reflections
2006 independent reflections
1896 reflections with *I* > 2σ(*I*)
*R*_int_ = 0.029

#### Refinement

*R*[*F* ^2^ > 2σ(*F* ^2^)] = 0.026
*wR*(*F* ^2^) = 0.067
*S* = 1.11
2006 reflections
106 parameters
3 restraints
H atoms treated by a mixture of independent and constrained refinement
Δρ_max_ = 0.47 e Å^−3^
Δρ_min_ = −0.61 e Å^−3^

### 

Data collection: *CrystalClear* (Rigaku, 2005 ▶); cell refinement: *CrystalClear*; data reduction: *CrystalClear*; program(s) used to solve structure: *SHELXS97* (Sheldrick, 2008 ▶); program(s) used to refine structure: *SHELXL97* (Sheldrick, 2008 ▶); molecular graphics: *SHELXTL* (Sheldrick, 2008 ▶); software used to prepare material for publication: *PRPKAPPA* (Ferguson, 1999 ▶).

## Supplementary Material

Crystal structure: contains datablocks I, global. DOI: 10.1107/S160053681001785X/fb2196sup1.cif

Structure factors: contains datablocks I. DOI: 10.1107/S160053681001785X/fb2196Isup2.hkl

Additional supplementary materials:  crystallographic information; 3D view; checkCIF report

## Figures and Tables

**Table 1:** Hydrogen-bond geometry (Å, °)

*D*—H⋯*A*	*D*—H	H⋯*A*	*D*⋯*A*	*D*—H⋯*A*
N2—H2*A*⋯O1	0.86	1.87	2.724 (4)	172
N4—H4*A*⋯I1^i^	0.86	2.95	3.764 (3)	159
N4—H4*B*⋯I1^ii^	0.86	3.20	3.758 (3)	125
N6—H6*A*⋯I1^iii^	0.86	2.88	3.647 (3)	149
N6—H6*B*⋯N1^iv^	0.86	2.15	3.009 (3)	173
O1—H1*A*⋯I1^v^	0.83 (2)	3.13 (4)	3.760 (4)	134 (5)
O1—H1*A*⋯I1^vi^	0.83 (2)	3.39 (5)	3.778 (3)	112 (4)
O1—H1*B*⋯I1	0.82 (2)	3.00 (3)	3.732 (4)	150 (5)
N5—H5*A*⋯N3^vii^	0.86	2.15	3.013 (4)	177
N5—H5*B*⋯I1^v^	0.86	2.97	3.698 (3)	143

Symmetry codes: (i) 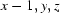; (ii) 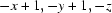; (iii) 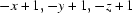; (iv) 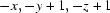; (v) 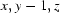; (vi) 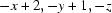; (vii) 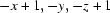.

**Fig. 1.**
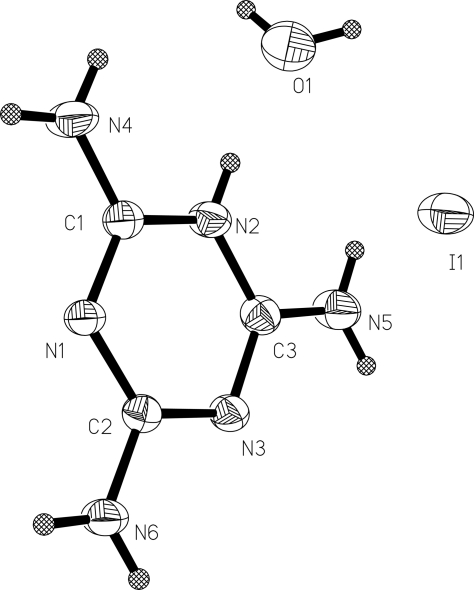
The title molecules with the atomic numbering scheme. The displacement ellipsoids are drawn at the 30% probability level.

**Fig. 2.**
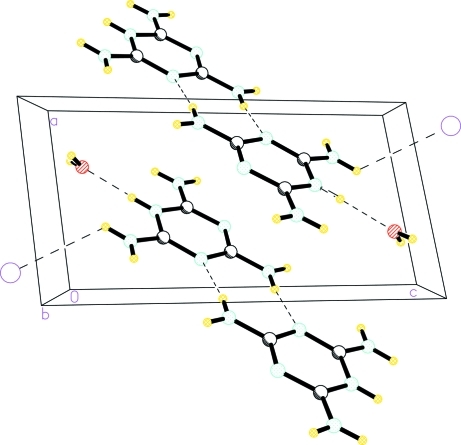
A view of the structure along the b axis. The dashed lines depict the hydrogen bonds.

## supplementary crystallographic information

### Comment

The melamine molecule and its organic and inorganic complexes or salts can develop supramolecular structures *via* multiple hydrogen-bonding systems by self-assembly of components which contain abundant hydrogen-bonding sites (Janczak *et al.*, 2001; Athikomrattanakul *et al.*, 2007). The present study is a part of systematic investigation of ferroelectric materials (Fu *et al.*, 2009; Hang *et al.*, 2009) that include metal-organic coordination compounds with organic ligands or are related to the structures with both organic and inorganic building fragments.

The compound was characterized by the X-ray powder diffraction (XRPD) at room temperature. The pattern calculated from the single-crystal X-ray data was in a good agreement with the observed at to the peak positions but with different peak intensities.

The structure is composed of the melaminium cations, iodide anions and the water molecules (Fig. 1). The melaminium cation is protonated at only one melamine site. The six-membered melaminium ring exhibits distortions from the regular hexagonal form. The internal C—N—C angle at the protonated N atom (119.5 (2)°) is greater than the other two C—N—C angles of the ring (115.5 (2)°) and the internal N—C—N angles involving the unprotonated ring N atoms (126.1 (2)°) are obviously larger than those containing protonated and unprotonated N atoms (121.4 (2)°) .

Fig. 2 shows a view down the *b* axis. The melaminium cations are interconnected by four N-H···N hydrogen bonds, forming ribbons parallel to (1 1 1). The water molecules connected by N-H···O hydrogen bonds (Tab. 1) form also a part of these ribbons. The ribbons are interconnected by other hydrogen bonds that involve I^-^ as well as by π-electron ring - π-electron ring interactions with the distance between the centroids of the neighbour melaminium rings (1-*x*,1-*y*,1-*z*) equal to 3.6597 (17) Å. The hydrogen bonds are summarized in Tab. 1. It is of interest that water oxygens despite of being quite close to each other are not interconnected by the hydrogen bond. The distance between the water oxygens is 2.9643 (40) Å [Symmetry code: 1-*x*, 1-*y*, -*z*]. The H atom of the protonated ring N atom (H2a) is donated to the water molecule, being involved in a strong N-H···O hydrogen bond. The other amine H atoms are involved in N—H···I and N—H···N hydrogen bonds. I^-^ anions that take part in the electrostatic equilibrium with the melaminium cations are also involved in O—H···I hydrogen bonds.

### Experimental

Melamine (0.252 g, 0.002 mol) was dissolved in 25 ml of water at 323 K. 0.569 g of 45% (weight concentration) solution of HI was added to the solution. (This amount corresponded to about 0.002 mol of pure HI.) The temperature was maintained at 323 K for one hour while stirring the mixture. Then the solution was let to cool down to room temperature. After several days, the title salt, C_3_H_7_N_6_^+^.I^-^.H_2_O, crystallized from the solution. The crystals were colourless, prismatic and of the average size about 0.2×0.3×0.4 mm.

### Refinement

All the hydrogens were discernible in the difference electron density maps. The positions of the H atoms of the melamine cations were refined using a riding model with N—H = 0.86 Å and *U*_iso_(H) = 1.2*U*_eq_(N). The coordinates of the water hydrogens have been refined under restrains 0.82 (2)Å; *U*_iso_(H) = 1.2*U*_eq_(O). (The constrained and the restrained values fit well to the trial refinement with the freely refined hydrogen parameters.)

Dielectric studies (capacitance and dielectric loss measurements) were performed on powder samples which have been pressed into tablets with conducting carbon glue deposited on their faces. The automatic impedance TongHui2828 Analyzer has been used (Uthrakumar *et al.*, 2008). In the measured temperature range from 80 to 450 K  (m.p. > 470 K), the temperature dependence of the relative permittivity at 1 MHz varied smoothly from 3.9 to 5.2 in the title compound. No dielectric anomaly has been observed.

### Crystal data

**Table:**

C_3_H_7_N_6_^+^·I^−^·H_2_O	*Z* = 2
*M**_r_* = 272.06	*F*(000) = 260
Triclinic, *P*1	*D*_x_ = 2.050 Mg m^−^^3^
Hall symbol: -P 1	Mo *K*α radiation, λ = 0.71073 Å
*a* = 6.0655 (12) Å	Cell parameters from 4510 reflections
*b* = 7.0370 (14) Å	θ = 3.2–27.5°
*c* = 11.413 (2) Å	µ = 3.59 mm^−^^1^
α = 104.02 (3)°	*T* = 293 K
β = 93.95 (3)°	Prism, colourless
γ = 109.08 (3)°	0.40 × 0.30 × 0.20 mm
*V* = 440.80 (19) Å^3^	

### Data collection

**Table:**

Rigaku SCXmini diffractometer	2006 independent reflections
Radiation source: fine-focus sealed tube	1896 reflections with *I* > 2σ(*I*)
graphite	*R*_int_ = 0.029
Detector resolution: 13.6612 pixels mm^-1^	θ_max_ = 27.5°, θ_min_ = 3.2°
ω scans	*h* = −7→7
Absorption correction: multi-scan (*CrystalClear*; Rigaku, 2005)	*k* = −9→9
*T*_min_ = 0.285, *T*_max_ = 0.487	*l* = −14→14
4551 measured reflections	

### Refinement

**Table:**

Refinement on *F*^2^	Primary atom site location: structure-invariant direct methods
Least-squares matrix: full	Secondary atom site location: difference Fourier map
*R*[*F*^2^ > 2σ(*F*^2^)] = 0.026	Hydrogen site location: difference Fourier map
*wR*(*F*^2^) = 0.067	H atoms treated by a mixture of independent and constrained refinement
*S* = 1.11	*w* = 1/[σ^2^(*F*_o_^2^) + (0.0285*P*)^2^ + 0.1083*P*] where *P* = (*F*_o_^2^ + 2*F*_c_^2^)/3
2006 reflections	(Δ/σ)_max_ = 0.008
106 parameters	Δρ_max_ = 0.47 e Å^−^^3^
3 restraints	Δρ_min_ = −0.60 e Å^−^^3^
30 constraints	

### Special details

**Table:**

Geometry. All esds (except the esd in the dihedral angle between two l.s. planes) are estimated using the full covariance matrix. The cell esds are taken into account individually in the estimation of esds in distances, angles and torsion angles; correlations between esds in cell parameters are only used when they are defined by crystal symmetry. An approximate (isotropic) treatment of cell esds is used for estimating esds involving l.s. planes.
Refinement. Refinement of *F*^2^ against ALL reflections. The weighted *R*-factor wR and goodness of fit *S* are based on *F*^2^, conventional *R*-factors *R* are based on *F*, with *F* set to zero for negative *F*^2^. The threshold expression of *F*^2^ > σ(*F*^2^) is used only for calculating *R*-factors(gt) etc. and is not relevant to the choice of reflections for refinement. *R*-factors based on *F*^2^ are statistically about twice as large as those based on *F*, and *R*- factors based on ALL data will be even larger.

### Fractional atomic coordinates and isotropic or equivalent isotropic displacement parameters (Å^2^)

**Table:**

	*x*	*y*	*z*	*U*_iso_*/*U*_eq_	
N1	0.1770 (4)	0.3970 (4)	0.3846 (2)	0.0392 (5)	
N2	0.4296 (4)	0.2565 (4)	0.2773 (2)	0.0433 (5)	
H2A	0.5039	0.2463	0.2159	0.052*	
N3	0.3428 (4)	0.1720 (4)	0.4600 (2)	0.0402 (5)	
N4	0.2758 (6)	0.4725 (5)	0.2059 (3)	0.0567 (7)	
H4A	0.1912	0.5504	0.2121	0.068*	
H4B	0.3504	0.4576	0.1449	0.068*	
N6	0.1007 (5)	0.3160 (4)	0.5631 (2)	0.0510 (6)	
H6A	0.1161	0.2533	0.6175	0.061*	
H6B	0.0144	0.3925	0.5713	0.061*	
O1	0.6721 (6)	0.1912 (5)	0.0884 (3)	0.0817 (9)	
H1A	0.707 (10)	0.091 (5)	0.051 (5)	0.123*	
H1B	0.731 (10)	0.301 (5)	0.070 (5)	0.123*	
C1	0.2911 (5)	0.3752 (4)	0.2904 (3)	0.0401 (6)	
C2	0.2097 (5)	0.2947 (4)	0.4671 (2)	0.0375 (5)	
C3	0.4499 (5)	0.1533 (4)	0.3622 (3)	0.0414 (6)	
I1	0.89281 (4)	0.76920 (3)	0.134131 (16)	0.05182 (10)	
N5	0.5833 (5)	0.0365 (5)	0.3468 (3)	0.0562 (7)	
H5A	0.6010	−0.0276	0.4000	0.067*	
H5B	0.6525	0.0246	0.2835	0.067*	

### Atomic displacement parameters (Å^2^)

**Table:**

	*U*^11^	*U*^22^	*U*^33^	*U*^12^	*U*^13^	*U*^23^
N1	0.0500 (13)	0.0449 (12)	0.0330 (11)	0.0251 (10)	0.0130 (10)	0.0164 (10)
N2	0.0505 (13)	0.0533 (13)	0.0369 (12)	0.0275 (11)	0.0152 (10)	0.0170 (11)
N3	0.0489 (12)	0.0450 (12)	0.0339 (12)	0.0243 (10)	0.0076 (10)	0.0133 (10)
N4	0.0751 (18)	0.0785 (19)	0.0448 (15)	0.0473 (16)	0.0294 (14)	0.0350 (14)
N6	0.0732 (17)	0.0630 (16)	0.0401 (13)	0.0427 (14)	0.0239 (12)	0.0264 (12)
O1	0.093 (2)	0.086 (2)	0.080 (2)	0.0379 (18)	0.0513 (17)	0.0311 (18)
C1	0.0445 (14)	0.0427 (14)	0.0369 (14)	0.0182 (12)	0.0088 (11)	0.0136 (11)
C2	0.0415 (13)	0.0382 (13)	0.0330 (13)	0.0148 (11)	0.0054 (11)	0.0095 (11)
C3	0.0424 (14)	0.0453 (14)	0.0375 (14)	0.0186 (12)	0.0035 (11)	0.0099 (12)
I1	0.06273 (16)	0.06633 (16)	0.03972 (14)	0.03312 (12)	0.01791 (10)	0.02193 (11)
N5	0.0674 (17)	0.0751 (18)	0.0485 (16)	0.0474 (15)	0.0192 (13)	0.0240 (14)

### Geometric parameters (Å, °)

**Table:**

N1—C1	1.322 (3)	N4—H4B	0.8600
N1—C2	1.357 (3)	N6—C2	1.321 (4)
N2—C1	1.357 (3)	N6—H6A	0.8600
N2—C3	1.366 (4)	N6—H6B	0.8600
N2—H2A	0.8600	O1—H1A	0.831 (18)
N3—C3	1.330 (4)	O1—H1B	0.822 (18)
N3—C2	1.354 (3)	C3—N5	1.321 (4)
N4—C1	1.325 (4)	N5—H5A	0.8600
N4—H4A	0.8600	N5—H5B	0.8600
			
C1—N1—C2	115.6 (2)	N1—C1—N4	120.5 (3)
C1—N2—C3	119.5 (2)	N1—C1—N2	121.9 (2)
C1—N2—H2A	120.3	N4—C1—N2	117.6 (3)
C3—N2—H2A	120.3	N6—C2—N3	117.0 (2)
C3—N3—C2	115.5 (2)	N6—C2—N1	117.0 (2)
C1—N4—H4A	120.0	N3—C2—N1	126.1 (2)
C1—N4—H4B	120.0	N5—C3—N3	120.1 (3)
H4A—N4—H4B	120.0	N5—C3—N2	118.5 (3)
C2—N6—H6A	120.0	N3—C3—N2	121.4 (2)
C2—N6—H6B	120.0	C3—N5—H5A	120.0
H6A—N6—H6B	120.0	C3—N5—H5B	120.0
H1A—O1—H1B	115 (3)	H5A—N5—H5B	120.0

### Hydrogen-bond geometry (Å, °)

**Table:**

*D*—H···*A*	*D*—H	H···*A*	*D*···*A*	*D*—H···*A*
N2—H2A···O1	0.86	1.87	2.724 (4)	172
N4—H4A···I1^i^	0.86	2.95	3.764 (3)	159
N4—H4B···I1^ii^	0.86	3.20	3.758 (3)	125
N6—H6A···I1^iii^	0.86	2.88	3.647 (3)	149
N6—H6B···N1^iv^	0.86	2.15	3.009 (3)	173
O1—H1A···I1^v^	0.83 (2)	3.13 (4)	3.760 (4)	134 (5)
O1—H1A···I1^vi^	0.83 (2)	3.39 (5)	3.778 (3)	112 (4)
O1—H1B···I1	0.82 (2)	3.00 (3)	3.732 (4)	150 (5)
N5—H5A···N3^vii^	0.86	2.15	3.013 (4)	177
N5—H5B···I1^v^	0.86	2.97	3.698 (3)	143

Symmetry codes: (i) *x*−1, *y*, *z*; (ii) −*x*+1, −*y*+1, −*z*; (iii) −*x*+1, −*y*+1, −*z*+1; (iv) −*x*, −*y*+1, −*z*+1; (v) *x*, *y*−1, *z*; (vi) −*x*+2, −*y*+1, −*z*; (vii) −*x*+1, −*y*, −*z*+1.
